# The colonization with *Candida* species is more harmful in the second trimester of pregnancy

**DOI:** 10.1007/s00404-017-4331-y

**Published:** 2017-03-03

**Authors:** Iris Holzer, Alex Farr, Herbert Kiss, Michael Hagmann, Ljubomir Petricevic

**Affiliations:** 1 Division of Obstetrics and Fetomaternal Medicine, Department of Obstetrics and Gynecology, Medical University of Vienna, Waehringer Guertel 18-20, Vienna, 1090 Austria; 2 Section for Medical Statistics (IMS), Center of Medical Statistics, Informatics and Intelligent Systems, Medical University of Vienna, Vienna, Austria

**Keywords:** Vaginal smear, Candidosis, *Candida* colonization, Preterm birth, Birthweight

## Abstract

**Purpose:**

Vaginal colonization with *Candida* species (spp.) during pregnancy has been associated with impaired pregnancy outcomes. There is a reduction in spontaneous preterm birth among women with recurrent asymptomatic colonization of *Candida* who were treated with clotrimazole. This study aimed to evaluate the impact of the trimester of vulvovaginal colonization with *Candida* species.

**Methods:**

Data from all women, who were tested positive for the vaginal colonization with *Candida* spp. during the first or second trimester of pregnancy, and who registered for a planned birth at our tertiary referral center between 2005 and 2014 were retrospectively analyzed. Their preterm birth rate served as the primary outcome variable. Secondary outcome variables were neonatal birthweight and Apgar score.

**Results:**

Overall, 1066 women were eligible for the study. In 673 women (63%), who were diagnosed with *Candida* spp. during the first trimester of pregnancy, the rate of preterm birth was 10% (*N* = 64). In 393 women (37%), who were diagnosed with candidosis during the second trimester, the preterm birth rate was 18% (*N* = 71; *p* = 0.0002). Neonates of women, who presented with vulvovaginal candidosis during the first trimester, had a mean birthweight of 3243 g, compared to 2989 g in the group with a second trimester colonization (*p* < 0.0001).

**Conclusion:**

Women who are colonized with *Candida* spp. during the second trimester of pregnancy have higher rates of preterm birth and lower neonatal birthweight than those who are colonized during the first trimester of their pregnancy. Screening programs for asymptomatic *Candida* colonization should take this information into account.

## Introduction

Preterm birth (PTB) is defined as birth before 37 completed gestational weeks. Despite all efforts in modern maternity care, PTB remains one of the most fundamental challenges in obstetrics, and the leading cause of neonatal morbidity and mortality [1]. Preterm neonates are at an increased risk for a wide range of adverse neonatal outcomes, in addition to the burden of considerable economic consequences that they cause for affected families and health services [2]. Although PTB is considered a multi-factorial event, vaginal infections remain the major associated factor in up to 40% of all cases with PTB [3–5].

According to Leli et al. [6], the colonization with *Candida* spp. occurs more frequently in pregnant compared to non-pregnant women (31.4 vs. 19.9%). In particular, pregnant women are more often suffering from asymptomatic *Candida* spp. colonization than their non-pregnant counterparts (46.5 vs. 16.0%) [6]. Apart from bacterial vaginosis, which has extensively been studied for its effect on pregnancy outcomes, there is evidence that even an aerobic bacterial infection during pregnancy should be treated to significantly improve perinatal outcomes [7]. The data on the effect of the antenatal vaginal colonization with *Candida* are still limited [3, 6]. Cotch and colleagues found no association of candidosis with low birthweight and PTB [8]. In contrary, our recently published data demonstrated that the recurrent asymptomatic vaginal colonization with *Candida* spp. in early pregnancy is indeed associated with PTB and low birthweight [9]. This stands in accordance with the results of a study that reported a reduction in spontaneous PTB among women with asymptomatic colonization of *Candida* spp. who were treated with clotrimazole [10].

Essentially, these results indicate that screening programs for the asymptomatic vaginal colonization with *Candida* spp. could be of value. However, more data about the ideal time-point and setting of screening programs are needed. To date, it remains unclear whether the primary colonization with *Candida* during the first or second trimester of pregnancy is more harmful. Our study is the first to evaluate the impact of the trimester of vulvovaginal candidosis in terms of pregnancy outcomes and PTB in particular.

## Patients and methods

Between 1 January 2005 and 1 January 2014, data from all women with singleton pregnancies, who were diagnosed with the vaginal colonization of *Candida* spp. at the Medical University of Vienna, Department of Obstetrics and Gynecology, were retrospectively analyzed. We considered vaginal smears of both symptomatic and asymptomatic women, who had a first-time *Candida* colonization during their first or second trimester of pregnancy. Eligible women had singleton pregnancies and registered for a planned birth at our obstetrical center. All of them were antenatally diagnosed with *Candida* spp. on the basis of their vaginal smears, which were undertaken either during the first or second trimester of pregnancy. Women with bacterial vaginosis, intermediate vaginal flora or other bacterial infections were excluded from the analyses. The standard treatment for *Candida* vulvovaginitis was local clotrimazole 0.1 g for 6 days within 3–5 days after diagnosis.

The cut-off between the first and second trimester of pregnancy was determined at 14 + 0 gestational weeks with the second trimester being defined as the gestational age between 14 + 0 and 28 + 6 weeks. Study group 1 consisted of women, who were primarily diagnosed with *Candida* colonization or candidosis during the first trimester, whereas Study group 2 consisted of women, who were primarily diagnosed with *Candida* colonization or candidosis during the second trimester of their pregnancy. Pregnancy outcomes were assessed on the basis of gestational age at birth, recorded as term birth at or later than 37 + 0 gestational weeks. PTB was defined as the spontaneous birth at or less than 36 + 6 gestational weeks. The rate of PTB served as the primary outcome measure. Secondary outcome measures included neonatal birthweight and the neonatal Apgar score. Stillbirth was defined as the term or preterm birth of a neonate that had died in utero and was born with an Apgar score of 0/0/0.

The demographic and background information was summarized and displayed using descriptive statistics. Discrete data are presented as *N* (%). The Chi-squared test was used to compare groups of categorical data. For comparison of continuous data, Welch’s *t* test was used. A two-sided *p* value <0.05 was considered statistically significant. Logistic and linear regression was used to determine the adjusted effect of variables. A multiple regression model was conducted that included the following potentially confounding variables that were unequally distributed between the groups and that were compared and considered to have an impact on the end-point: maternal age, tertiary education, history of PTB and nicotine abuse. All relevant data were collected from obstetric databases, patient charts and microbiologic reports.

## Results

Retrospective data analysis identified a total of 1066 women with singleton pregnancies, who were antenatally, and for the first time, diagnosed with asymptomatic and/or symptomatic *Candida* spp. on the basis of their vaginal smears, during the first or second trimester of their pregnancy. Out of these women, 673 (63%) women were diagnosed with *Candida* spp. during the first trimester, and 393 (37%) women were diagnosed during the second trimester of pregnancy. Overall, mean maternal age at the time of birth was 30.2 ± 6.3 years. Table 1 illustrates the baseline variables, sociodemographic characteristics and obstetrical history of the included study participants. No serious adverse events were reported for the treatment that was subsequently initiated.

**Table 1:** Baseline variables of the 1066 study participants with *Candida* colonization or candidosis

Variable	Baseline variables (*N* = 1066)		All
	Study group, trimester 1	Study group, trimester 2	
	Mean ± SD, *N* (%)	Mean ± SD, *N* (%)	Mean ± SD, *N* (%)
Participants	673 (63)	393 (37)	1066 (100)
Age at birth (years)	30.4 ± 6.0	29.8 ± 6.6	30.2 ± 6.3
Nicotine abuse	144 (21)	106 (27)	250 (23)
Academic education	45 (7)	34 (9)	23 (9.7)
History of PTB	9 (1)	9 (2)	18 (2)

*PTB* preterm birth, *SD* standard deviation, *N* number

In the overall study cohort, the mean gestational age at birth was 38.5 ± 3.0 weeks, corresponding to a mean birthweight of 3144 ± 694 g. In women of study group 1, the mean gestational age at birth was 38.9 ± 2.3 weeks, and in those of study group 2, it was 37.8 ± 3.9 gestational weeks. Infants of women in study group 1 had a mean birthweight of 3234 ± 600 g, compared to a mean of 2989 ± 810 g in those who were assigned to study group 2 (*p* < 0.0001). In study group 1, the rate of PTB was 10% (*N* = 64), compared to 18% (*N* = 71) in study group 2 (*p* = 0.0002). This was confirmed in a multiple regression model after adjustment for nicotine abuse as the only remaining significant confounder.

The mean Apgar scores at 1 min were significantly different for neonates of women of study group 1 vs. those of study group 2 (8.7 ± 1.0 vs. 8.4 ± 1.6; *p* = 0.0004). Table 2 summarizes the pregnancy outcomes of the 1066 study participants with *Candida* colonization.

**Table 2:** Obstetric outcomes of the 1066 study participants with *Candida* colonization or candidosis

Variable	Category/unit	Study group (*N* = 1066)		All	Test
		Trimester 1	Trimester 2		
		Mean ± SD, *N* (%)	Mean ± SD, *N* (%)	Mean ± SD, *N* (%)	*p* value
Pregnancy outcome	Live birth	672 (100)	388 (99)	1060 (99)	NS
	Stillbirth	1 (0)	5 (1)	6 (1)	
Prematurity	Preterm birth	64 (10)	71 (18)	135 (13)	0.0002
	No preterm birth	609 (90)	322 (82)	931 (87)	
Apgar at 1 min	Score	8.7 ± 1.0	8.4 ± 1.6	8.6 ± 1.3	0.0004
Apgar at 5 min	Score	9.7 ± 0.9	9.4 ± 1.6	9.6 ± 1.2	NS
Apgar at 10 min	Score	9.8 ± 1.1	9.6 ± 1.6	9.7 ± 1.3	NS
Gestational age at birth	Weeks	38.9 ± 2.3	37.8 ± 3.9	38.5 ± 3.0	NS
Birthweight	Grams	3234 ± 600	2989 ± 810	3144 ± 694	<0.0001

*N* number, *SD* standard deviation, *NS* not significant

## Discussion

The present study was undertaken to evaluate the impact of the trimester of *Candida* colonization with regard to pregnancy outcomes. As it is known that the recurrent asymptomatic colonization with *Candida* is involved in the multifactorial event of PTB, we now wanted to evaluate the impact of vaginal colonization or infection with *Candida* spp. on subsequent pregnancy outcomes. This information could help to determine the ideal time-point for a routine antenatal infection-screening program.

It has already been demonstrated that routine antenatal infection-screening programs have the potential to reduce the rates of PTB and late miscarriage in a general population of pregnant women [1]. For this reason, our department has integrated a program for all pregnant women, to screen and treat for the asymptomatic colonization with pathogens, including bacterial vaginosis and vaginal candidosis [11]. As it is known that recurrent vulvovaginal candidosis can be harmful during early pregnancy, the need for early detection and adequate antifungal treatment is clearly emphasized [10].

A recently published review reported that the use of locally delivered dequaliniumchloride led to a complete cure in 84% of the pregnant women with *Candida* [12]. Another treatment of vaginitis in pregnancy is octenidine dihydrochloride/phenoxyethanol that has shown a positive effect in women with bacterial vaginosis and/or candidosis [13]. More easily, screening strategies prior to any infection could help balancing the health care budget, as shown in a cost-effectiveness analysis [14]. The costs per prevented PTB or pregnant woman screened amounted to merely 7% of the direct costs saved as a result of screening. Indeed, PTB incurs notable social and healthcare costs, particularly in European countries with national health care systems and socialized medicine. The antenatal infection-screening program that we used in our study has been proven to reduce PTB rates by 50%, and therewith save these costs [14, 15].

In addition to the socioeconomic advantages, the high prevalence of *Candida* colonization guided us to integrate an antenatal infection-screening program during routine pregnancy care. Data showed that patients with recurrent candidosis during early pregnancy had higher rates of PTB [9]. In the present study, we only analyzed data of women, who were uniformly colonized or affected with *Candida* spp. and in this patient group, we found an overall PTB rate of 13%. This rate seems to be higher than the PTB rate in a large retrospective Hungarian study that shows a 4.6% rate of PTB in women, who were colonized with *Candida* spp. and treated with clotrimazole [16]. The reason for the higher PTB rate in our study might be that our study was conducted in the tertiary setting, including women with high-risk pregnancies and comorbidities that might interfere with the susceptibility for vaginal candidosis. In contrast, the Hungarian study was a nationwide trial that contained mostly data of women with low-risk pregnancies. A randomized controlled trial from Australia [17] reported a PTB rate of 4% in pregnant women with asymptomatic vaginal *Candida* colonization who were treated with clotrimazole. Considering the comparable PTB rates among Austrian and Australian women [18, 19], the 4% PTB rate among the Australian women with candidosis seems to be actually lower than that of our study. The prospective design of the Australian study could have led to the recruitment and randomization of patients without other risks for PTB, since the authors of this study did not clearly list their exclusion criteria.

In 2008, a prospective study analyzed the prevalence of *Candida* spp. in pregnant women and reported that the highest colonization rate (65.7%) was found in women between 21 and 30 years of age [20]. The mean age in our study population was 30.2 years, which seems to be comparable on the basis of our tertiary setting. The mean birthweight of all our study participants with *Candida* colonization was 3144 g at a mean gestational age at birth of 38.5 gestational weeks. This seems to be comparable to the mean birthweight of 3330 g in the nationwide Hungarian trial [16]. Here, the authors reported a mean gestational age of 39.8 weeks at birth, which might be explained by the higher rate of cesarean sections in our tertiary referral center.

The main finding of our study was that the primary colonization with *Candida* spp. was more harmful in the second than in the first trimester of pregnancy. This finding was highly statistically significant in a multiple regression model. In a large prospective study from Belgium, it was demonstrated that women with a normal vaginal flora in the first trimester of pregnancy had a 75% lower risk of preterm birth before 35 + 0 weeks of gestation, when being compared to women with an abnormal flora [21]. In the meanwhile, the PTB-risk increasing effect of bacterial vaginosis and recurrent *Candida* colonization during the first trimester of pregnancy was described [9]. The finding of our study, that the occurrence of *Candida spp*. during the second trimester of pregnancy also influences pregnancy outcomes, and that it is even more harmful during the second trimester of pregnancy, has not yet been described in the literature. It seems to be of particular interest for the planning and organization of infection screening-programs aiming to detect vulvovaginal candidosis, even after first trimester of pregnancy.

According to a recent study on vulvovaginitis in pregnant women, only 7% of infections are occurring in the first, but 60% in the second trimester [22]. However, the data of this study included a variety of pathological conditions, and not only the colonization with *Candida*. Theoretically, immunologic alternations during pregnancy could have contributed to the altered severity and susceptibility of pathogen colonization during pregnancy. Aspects of innate immunity are enhanced towards late pregnancy, so that this may contribute to an immune down-regulation of the physiologic lower genital tract [23]. This could be one of the reasons for the increased detection of vaginal infections during the second trimester of pregnancy. To prevent PTB, Roberts et al. [17] decided to screen and treat for candidosis in the early to mid second trimester of pregnancy. We suggest that this is the ideal time-point for an antenatal infection-screening program.

It should clearly be stated that our study has a number of limitations. Other factors that occurred after the screening could have contributed to the reported pregnancy outcomes. Moreover, we did not distinguish between moderate and heavy colonization in our study, as Roberts et al. [17] did it in their large controlled trial. Furthermore, we were not able to perform PCR methods. We have to admit that this was not possible due to the retrospective design of our study, which clearly limits its value.

We are also aware that it would have been ideal to differentiate between different *Candida* spp., as it was done in some previous studies [16, 17]. However, we know that *C. albicans* is the most frequent species in 80–90% of all women who suffer from candidosis [20].

## Conclusion

In this study, we have demonstrated that women who are colonized with *Candida* spp. during the second trimester of pregnancy have higher rates of preterm birth and lower neonatal birthweight than those who are colonized during the first trimester of their pregnancy. Despite all limitations, our findings lend support to the idea that time-point of *Candida* diagnosis and treatment matters. As recurrent candidosis is known to increase the risk of PTB, our results indicate that the antenatal screening for *Candida* should ideally be performed during the early second trimester of pregnancy. We suggest there is a need for adequate infection screening programs in routine pregnancy care, which should take this information into account.
